# The persistent effects of foetal growth on child and adolescent mental health: longitudinal evidence from a large population-based cohort

**DOI:** 10.1007/s00787-022-02045-z

**Published:** 2022-07-21

**Authors:** Niamh Dooley, Colm Healy, David Cotter, Mary Clarke, Mary Cannon

**Affiliations:** 1 Department of Psychiatry, Royal College of Surgeons in Ireland, Dublin, Ireland; 2 Trinity College Institute of Neuroscience, Trinity College Dublin, Dublin, Ireland; 3 Department of Psychology, Royal College of Surgeons in Ireland, Dublin, Ireland; 4 Department of Psychiatry, Beaumont Hospital, Dublin, Ireland

**Keywords:** Birth weight, Foetal growth, Internalizing, ADHD, Developmental psychopathology, Sex differences

## Abstract

Low birth weight for one’s gestational age is associated with higher rates of child psychopathology, however, most studies assess psychopathology cross-sectionally. The effect of such foetal growth restriction appears to be strongest for attention problems in childhood, although adult studies have found associations with a range of outcomes, from depression to psychosis. We explore how associations between foetal growth and psychopathology change across age, and whether they vary by sex. We used a large nationally representative cohort of children from Ireland (*N* ~ 8000). Parents completed the Strengths and Difficulties Questionnaire (SDQ) at 3 time points (age 9, 13 and 17). Outcomes included a total problems scale and subscales measuring attention/hyperactivity, peer, conduct and emotional problems. Foetal growth had significant associations with all problem scales, even after controlling for sex, socioeconomic factors and parental mental health. The magnitude of these effects was small but relatively stable across ages 9–17. In males, foetal growth had the strongest associations with attention/hyperactivity and peer problems, whereas females showed more widespread associations with all four subscales. There was a trend for the association between foetal growth and emotional problems to increase with advancing age, approaching the borderline-abnormal threshold by age 17. Reduced foetal growth predicted persistently higher scores on all measured aspects of child and adolescent psychopathology. Associations with child attention/hyperactivity may generalize to a wider array of adult psychopathologies via adolescent-onset emotional problems. Future studies should explore potential age-dependent effects of foetal growth into the early 20s.

**Supplementary Information:**

The online version contains supplementary material available at 10.1007/s00787-022-02045-z.

## Introduction

Low birth weight (BW), either due to premature birth or restricted foetal growth, has been associated with subsequent mental health difficulties (meta-analyses [1, 8, 26]). One explanation for this phenomenon is the “developmental origins of adult health and disease hypothesis” which posits that the foetus permanently adjusts or programmes certain aspects of its physiology based on the intra-uterine environment. While such alterations may be adaptive in the short-term and improve chances of neonatal survival, they may become maladaptive in the long-term, increasing susceptibility to physical or mental illnesses later in life [18]. Adult psychiatric disorders linked with lower BW have included depression [11], psychotic disorders [2, 9] and substance-use disorders [30]; however, the most reliable psychological correlates of BW *in children* appear to be attention-deficit/hyperactivity disorder (ADHD) and autism [1, 9, 12, 31].

Most studies on the association between BW and mental health have assessed the latter cross-sectionally; however, several longitudinal studies suggest that the psychological effects of BW may change throughout development. Lærum et al. [19] assessed the mental health of 3 groups—very low birth weight (VLBW), small-for-gestational age (SGA) and a control group—across ages 14, 19 and 26 years and found that VLBW and SGA groups had higher rates of mental illness compared to controls, with the difference in risk increasing with age. For example, those born SGA, had an increased probability of any disorder at age 14 (9% SGA vs 5% controls), a disparity that widened by age 19 (25% vs. 8%) and again at age 26 (38.5% vs 13%). Another study found that number of depressive and anxiety symptoms were similar for extremely low birth weight (ELBW) individuals and controls at age 15, but diverged thereafter, increasing in ELBW individuals and decreasing in controls over the subsequent 20 years [38]. Similarly, Husby et al. [17] found that, in a narrower age-range from 20 to 23 years, internalizing and general mental health problems increased linearly in VLBW individuals while remaining relatively unchanged in normal BW controls. Taken together, these studies suggest that there may be an age-dependent association between BW and mental health in general (and internalizing symptoms in particular) during the transition from childhood to adulthood. We therefore hypothesize that the association between BW and internalizing symptoms grows stronger between the ages of 9 and 17 years.

Attention-deficit/hyperactivity disorder (ADHD) and its symptoms have been an extensively studied psychological consequence of low BW, however there is mixed evidence that the association persists into adolescence. In support of a stable and persistent association, a meta-analysis showed that age did not moderate the association [26] and cross-sectional studies suggest attention deficits linked with low BW and SGA persist until adulthood [5, 24, 36, 37]. Further, twin studies show that the lower BW twin continues to report higher ADHD symptoms up to the mid-teens [16, 23]. In support of a weakening association over time, Hultman et al. [16] found the continuous association between BW and ADHD symptoms among twins was significant at 8–9 years but not at 13–14 years, and Lim, et al. [23] found BW had a decreasing association with ADHD symptoms from ages 8 to 16. The potential age-dependency of this association therefore requires further exploration.

Lower BW is often assumed to affect males and females in a similar manner however there is both animal and human evidence to the contrary. In rats, foetal growth restriction and prenatal anoxia has sex-specific effects on neurotransmitter function and behaviour [21, 22]. In 9–10-year-old children from the U.S. the inverse associations between BW and attention problems, aggressive behaviours and general psychopathology were driven largely by males [12], and in an independent sample of 6–19-year-old children, the same was found for ADHD and externalizing symptoms [27]. However, a meta-analysis found the association between BW and ADHD symptoms did not vary significantly by sex [26] raising questions about the generalizability of such findings. In an effort to bridge these inconsistent findings, we explore whether males and females differ in the *longitudinal* effects of BW.

BW is closely related to gestational age at birth such that infants born premature are likely smaller. The “appropriateness” of BW for an infant’s gestational age may therefore be a more useful measure of the quality of growth in utero, often referred to as “foetal growth”. The association between foetal growth and attention problems has been shown to be independent of gestational age [15, 26, 32], therefore foetal growth is the focus exposure of this study. No study to our knowledge has explored the associations between common deviations in gestational age (e.g., preterm; < 37 weeks) and longitudinal mental health in a large population cohort. Therefore, we do so as a secondary aim.

We investigated associations between foetal growth and psychopathology in a large population-based cohort at ages 9, 13 and 17. Our primary aim was to explore the age-related changes in the association between foetal growth and psychopathology, controlling for gestational age and potentially confounding socioeconomic and familial factors. We hypothesized that the association would strengthen over time for emotional (internalizing) problems. As secondary aims, we explored longitudinal effects of gestational age, and sex differences in the longitudinal effects of foetal growth.

## Methods

### Participants

Growing Up in Ireland (GUI) is an ongoing Irish Government-funded study of children which follows over 8000 children (born 1997–1998). Initially, primary schools were chosen to approximate national distribution of schools based on geographical region, disadvantaged status, co-ed status (boys, girls, mixed) and religious denomination. Children (and their parents) were invited to participate from chosen schools (see Acknowledgments for further details). Data collection began at age 9 (*N* = 8658) with follow-ups at ages 13 (*N* = 7423) and 17 (*N* = 6212). Descriptive statistics are provided for both weighted (Table S1) and unweighted (Table 1) data, which weights individuals based on their representativeness of the Irish population at the time and to compensate for attrition. We excluded all non-singleton born children (e.g., twins) from this study given systematic differences in foetal growth.

**Table 1:** Descriptive statistics

Categorical variables	Age 9		Age 13		Age 17	
	*N*	%	*N*	%	*N*	%
Males	4030	48.6%	3571	48.9%	2940	48.8%
Females	4263	51.4%	3727	51.1%	3086	51.2%
Premature born (< 37 w)	964	11.7%	829	11.5%	685	11.5%
Born late (> 41 w)	2038	24.8%	1813	25.0%	1474	24.7%
Single parenthood	964	12%	965	13%	896	15%
1 Parent with mental illness	1178	14%	874	12%	795	13%
2 Parents with mental illness	179	2%	190	3%	158	3%

Continuous variables	Range	M (SD)	Range	M (SD)	Range	M (SD)
BW (kg)	0.45–6.10	3.53 (0.57)	0.62–6.10	3.54 (0.56)	0.62–6.10	3.54 (0.56)
Household income (€)^a^	504–223 K	21 K (14 K)	549–134 K	18 K (10 K)	504–1.2 M	17 K (22 K)
Parental education level (1–6)	1–6	4.0 (1.3)	1–6	4.2 (1.3)	1–6	4.1 (1.3)
SDQ total problems	0–37	7.38 (5.02)	0–35	6.49 (5.05)	0–33	6.48 (4.93)
SDQ Attention/hyperactivity	0–10	2.98 (2.42)	0–10	2.54 (2.34)	0–10	2.23 (2.16)
SDQ emotional	0–10	2.01 (1.97)	0–10	1.78 (1.93)	0–10	1.94 (2.09)
SDQ peer	0–10	1.14 (1.43)	0–10	1.08 (1.44)	0–10	1.36 (1.46)
SDQ conduct	0–10	1.25 (1.42)	0–10	1.10 (1.38)	0–10	0.95 (1.27)

^a^Household income to the nearest thousand (K). Income deciles were used in the analysis to limit extreme values

## Materials/measures

### Strengths and difficulties questionnaire (SDQ)

The SDQ ([13]), which assesses mental and behavioural problems in children, was completed by the primary caregiver (most often the mother) at all 3 timepoints. The SDQ contains 25 items with response options on a 3-point Likert scale from “Not True” (0) to “Somewhat True” (1) to “Certainly True” (2). SDQ subscales include attention/hyperactivity, emotional, peer relationship, and conduct problems (each ranging 0–10). The prosocial behaviour scale was excluded from this study. A total problems score can be obtained by summing the 4 problem scores. We used cut-offs denoting “borderline” and “abnormal” scores as suggested by a survey of UK youths aged 4–17 (Fig. S1, [14]).

### Foetal growth

BW was ascertained by maternal recall when the child was aged 9. Maternal recall of BW 9 years after the birth has been found to align closely with medical records [33]. BW was treated as continuous (in kilograms) for most analysis but was split into 6 groups for sensitivity analysis with the following ranges: (1) < 2.50 kg (*N* = 315) (2) 2.50–2.99 kg (*N* = 913), (3) 3.0–3.49 kg (*N* = 2632), (4; reference) 3.5–3.99 kg (*N* = 2852), (5) 4.0–4.49 kg (*N* = 1161), (6) 4.5 kg or greater (*N* = 312). Gestational age at birth was categorical with 3 levels: on-time (37–41 weeks; reference group), early (36 weeks or less) and late (42 weeks or more). Foetal growth was approximated by the fixed effect of BW, controlling for gestational age.

### Covariates

#### Socioeconomic factors

Income, parental education and number of parents in household were recorded at each time point. Income referred to the family’s income equivalized to account for differences in size and composition of households (further detail in Supplementary Material) and income deciles were used to avoid outliers. Parental education referred to the highest education level of both parents (where relevant) from: (1) none/primary, (2) lower secondary, (3) higher secondary/technical vocational (4) non-degree certificate (5) primary degree (6) postgraduate degree. Single parenthood was captured by the primary caregiver responding “*No*” to the item “*Do you have a spouse/partner who lives here with you in the household?*”.

#### Parental psychiatric history

This variable combined 2 criteria. The first was “*Have you* [ever] *been treated by a medical professional for clinical depression, anxiety, “nerves” or phobias ever/since the last interview?*”. The second criterion was existence of an ongoing and chronic health problem that fell within the category of “Mental and Behavioural Disorders” (F00-F99) as defined by the International Classification of Diseases (ICD-10). If a parent met either, they were considered to have a history of mental illness. Parental psychiatric history was a treated as a continuous count of parents with a psychiatric history (range = 0–2). This variable was available for each time point thus could change over time.

### Statistics

To account for age-related change in SDQ scores, we ran both cross-sectional (1 linear model per age using *glm*) and longitudinal models (1 mixed model for all data using *glmer*) using the lme4 package in R [4]. Generalized linear models with a gamma distribution and identity link were run as SDQ outcomes were highly skewed (Fig. S1; [29]). A Bonferroni-corrected p value of 0.01 was used to identify significant findings (corrected for number of outcomes tested; 0.05/5).

A preliminary longitudinal model (Model 1 +) tested which interactions and polynomials should be included. We included several second order polynomials: BW^2^, as some studies have found both low and high BWs are linked with psychopathology [20], and time^2^, to allow for non-linear relationships between advancing age and psychopathology (e.g., peak problems at age 13). Interactions included BW × Time given this was our primary question, Time × Sex given descriptive plots showed these interactively influenced SDQ scores (Fig. S2), and BW × Sex, BW × Time × Sex and Time × Gestational Age given these were secondary questions. Only polynomials and interactions showing a significant effect on at least one SDQ outcome were included in the final Model 1. Model 2 additionally adjusted for socioeconomic factors while Model 3 adjusted for parental psychiatric history. Only the fully adjusted results (Model 3) are reported in main text. Others can be found in supplementary material.

All continuous predictors (BW, parental education, income) were mean-centred. For categorical predictors, the most common level became the reference. Contrast levels for sex were coded as 0.5 and − 0.5 (reference: female). Time was numeric and centred on age 13 as the midpoint of the study (i.e., time points coded − 1, 0, 1). Reported beta coefficients (B) are unstandardized, representing units of the SDQ. For interpretability, effect estimates are also expressed as a percentage of the total possible score. That is, if *B* = 0.50 for the association between BW and peer problems, then each kilogram drop of BW would be associated with a 5% (0.50/10) increase on this scale.

One sensitivity analysis explored non-linearities in the association between BW and SDQ scales. For this, BW was treated as a categorical variable with 6 levels in fully adjusted longitudinal models (Model 3). A second sensitivity analysis explored whether associations between BW and SDQ scales were significantly altered by successive adjustment for gestational age. For this we tested models without gestational age, controlling for main effects of gestational age, and controlling for interactive effects of BW and gestational age.

## Results

**Fig. 1:**
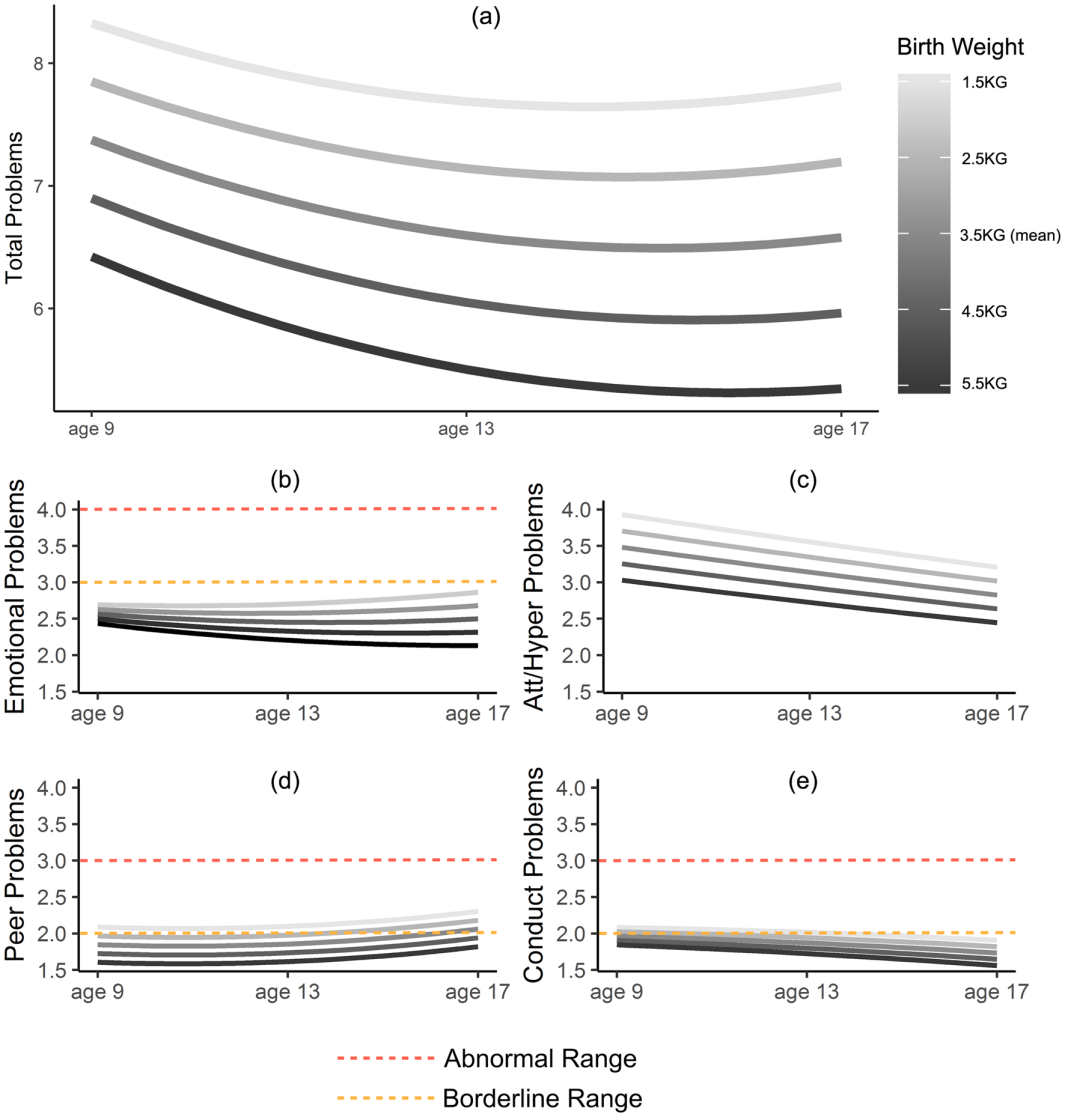
Predicted mental health trajectories from age 9–17 for various birth weights. Shown are trajectories of SDQ total problems (**a**), emotional problems (**b**), attention/hyperactivity problems (**c**), peer problems (**d**) and conduct problems (**e**). Adjusted for time, time^2^, sex, sex × time, gestational age at birth, socioeconomic factors and parental mental health. Borderline cut-offs out of range for (**a**) total problems (13) and (**c**) attention/hyperactivity problems (5)

BW ranged from 0.45 to 6.1 kg (mean = 3.5 kg) and 60% of children were born on-time (37–41 weeks’ gestation; Table 1). Attention/hyperactivity and conduct problems decreased linearly from age 9 to 17 (Fig. 1), with similar slopes in males and females, while emotional problems showed a strong sex difference after age 13 (Fig. S2).

### Longitudinal effects of BW

The association between BW and all SDQ scales was better described as linear than quadratic (Table S2) therefore the BW^2^ term was dropped from further analysis. A sensitivity analysis using BW groups verified there were no major non-linearities in associations with SDQ scales (Fig. S5). Figure 1 shows the predicted longitudinal trajectories of SDQ scores across ages 9–17 for five different BWs, along with the SDQ cut-points for abnormal and borderline-abnormal behaviour.

**Fig. 2:**
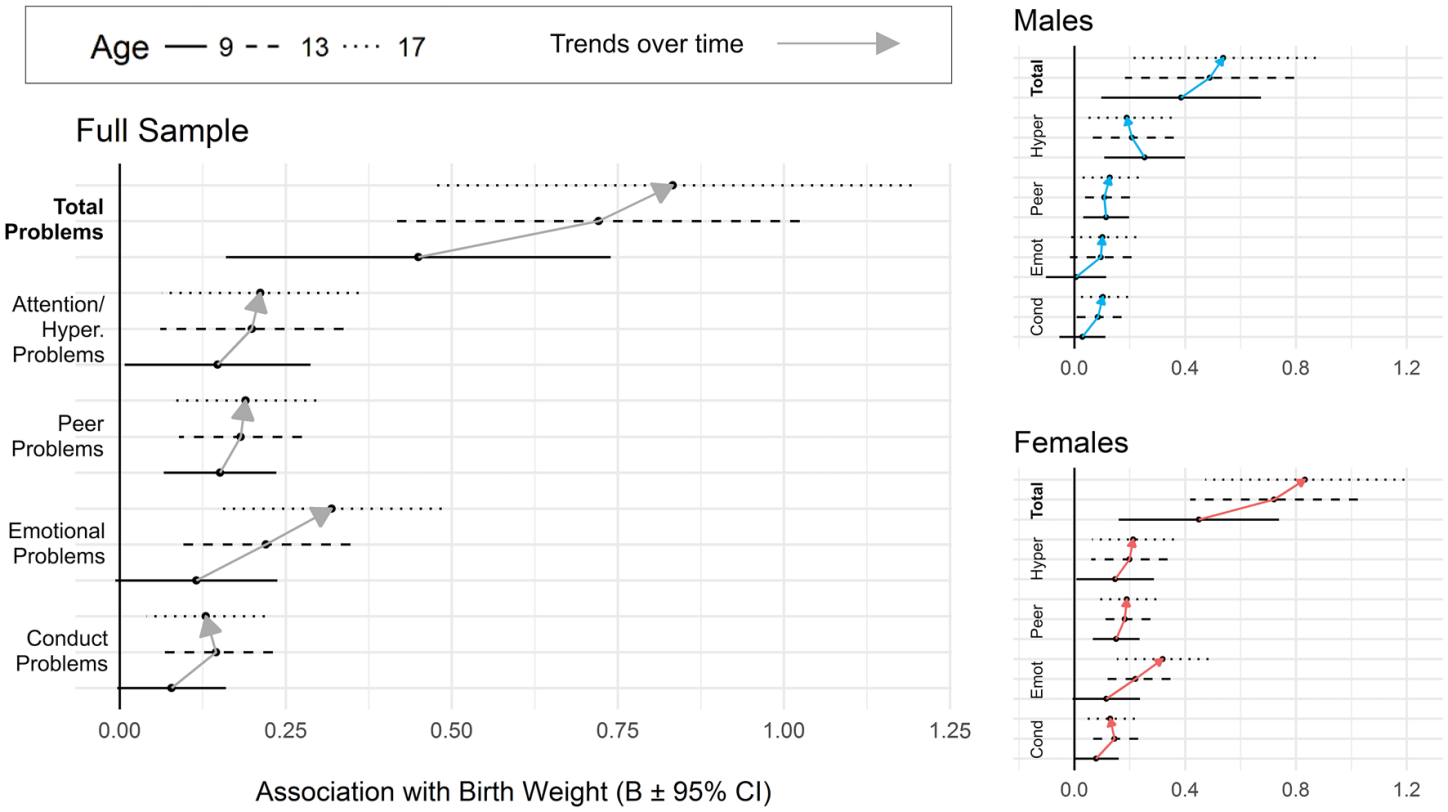
Association between BW and each SDQ scale at each time point (age 9, 13 and 17) in the full sample (left) and each sex separately (right). Association between BW and total problems is larger than subscales due to larger range of scores (0–40 vs 0–10). Association estimates were adjusted for sex, socioeconomic factors and parental mental health

Figure 2 shows that the association coefficient between BW and attention/hyperactivity problems was of similar magnitude (*B* ~ 0.20) at ages 9, 13 and 17. Consistently, longitudinal modelling showed that the main effect of BW on this scale was significant (*B* = − 0.21 [− 0.13, − 0.29], *t* = − 3.56, *p* < 0.001), while the interaction between BW and time on attention/hyperactivity was not (*B* = 0.02 [− 0.04, 0.08], *t* = 0.58, *p* = 0.57; Table 2). There was a relatively stable and persistent association between BW and *peer problems* across ages 9–17 (Fig. 2) which was reflected by a significant main effect of BW on peer problems longitudinally (*B* = − 0.12, SE = 0.02, *t* = − 5.79, *p* < 0.001) but lack of significant interaction between BW and time (Table 2).

**Table 2:** Cross-sectional and longitudinal effects of birth weight on SDQ scores from fully adjusted models

	Cross-sectional effects of birth weight^a^						Longitudinal effects^b^			
	Age 9		Age 13		Age 17		Main effect of birth weight		Interaction birth weight x Time	
	*B* (SE)	*t*	*B* (SE)	*t*	*B* (SE)	*t*	*B* (SE)	*t*	*B* (SE)	*t*
Total Problems	− 0.42 (0.10)	− 4.03**	− 0.61 (0.11)	− 5.56**	− 0.66 (0.13)	− 5.28**	− 0.55 (0.08)	− 7.14**	− 0.07 (0.06)	− 1.11
Attention/Hyper. Problems	− 0.19 (0.05)	− 3.78**	− 0.21 (0.05)	− 4.04**	− 0.20 (0.06)	− 3.64**	− 0.21 (0.04)	− 5.80**	0.02 (0.03)	0.58
Peer Problems	− 0.13 (0.03)	− 4.25**	− 0.14 (0.03)	− 4.25**	− 0.16 (0.04)	− 4.13**	− 0.12 (0.02)	− 5.79**	< 0.01 (0.02)	0.001
Emotional Problems	− 0.05 (0.04)	− 1.33	− 0.14 (0.04)	− 3.40**	− 0.17 (0.05)	− 3.35**	− 0.12 (0.03)	− 4.26**	− 0.06 (0.03)	− 2.32^†^
Conduct Problems	− 0.05 (0.03)	− 1.79	− 0.11 (0.03)	− 3.62**	− 0.12 (0.03)	− 3.54**	− 0.07 (0.02)	− 3.70**	− 0.01 (0.02)	− 0.83
Number of Observations	7476		6631		5285		19,416			

**p < 0.001 *p < 0.01 ^†^*p* < 0.05

^a^Adjusted for sex, gestational age at birth, household income, parent education, single-parenthood, parental psychiatric history

^b^Adjusted for sex, time, time^2^, sex *x* time, gestational age at birth, household income, parent education, single-parenthood, parental psychiatric history

The association between BW and emotional problems increased over time (Figs. 1b, 2). Cross-sectional estimates show that BW was not a significant predictor of emotional problems at age 9 but was at ages 13 and 17 (Table 2; fully adjusted model). In longitudinal models, the main effect of BW was significant (*B* = − 0.12 [− 0.06, − 0.18], *t* = − 4.26, *p* < 0.001) and the interaction of BW and time was significant, albeit at the uncorrected alpha level (*B* = − 0.06 [− 0.001, − 0.12], *t* = − 2.32, *p* = 0.02; Table 2). Our hypothesis was thus partially supported.

The main effect of BW on conduct problems was significant (*B* = − 0.07 [− 0.03, 0.11], *t* = − 3.70, *p* < 0.001) while the interaction of BW and time was not (Table 2). The total problem score showed a significant main effect of BW (*B* = − 0.55 [− 0.39, − 0.71], *t* = − 7.14, *p* < 0.001) but no significant interaction with time (*B* = − 0.07 [− 0.19, 0.05], *t* = − 1.11, *p* = 0.27; Table 2).

These main effects indicated that every kilogram drop in BW (all other factors held constant) was linked with a 2.1% increase in attention/hyperactivity problems, a 1.2% increase in peer and emotional problems, a 0.7% increase in conduct problems and a 1.3% increase in total problems.

BW was associated with variance in the “normal” range for total problems, emotional and attention/hyperactivity SDQ scales. However, any BW below the mean was associated with borderline peer problems by age 17, and low BW (< 2.5 kg) was associated with borderline conduct problems at age 9 only (Fig. 1).

### Sex differences

The 2-way interaction between BW and sex, and the 3-way interaction between BW, sex and time were not significant for any SDQ outcome (Table S2) so they were excluded from further models. Sex-stratified analyses were performed regardless, to compare sex-specific estimates.

Stronger associations between BW and attention/hyperactivity were observed in males (*B* = − 0.24, SE = 0.05, *t* = − 4.43, *p* < 0.001) compared to females (*B* = − 0.18, SE = 0.05, *t* = − 3.62, *p* < 0.001; Tables S8-9) in sex-stratified longitudinal analyses. Figure 2 suggests the BW by time trend on emotional problems, observed in the full sample, was driven by females. This was supported by sex-specific statistics: cross-sectional data showed BW was not significantly associated with emotional problems in males at any age, but that the association increased steadily in females from age 9 (*B* = − 0.12, SE = 0.06, n.s.), to age 13 (*B* = − 0.22, SE = 0.07, *p* < 0.001), to age 17 (*B* = − 0.32, SE = 0.08, *p* < 0.001; Tables S8-9). Similarly, longitudinal models showed a stronger BW × time interaction on emotional problems in females (*B* = − 0.08, SE = 0.04, *t* = − 1.77, *p* = 0.08) compared to males (*B* = − 0.05, SE = 0.03, *t* = − 1.47, *p* = 0.14) though neither was non-significant.

### Gestational age

Interactions between gestational age and time were not significant for any SDQ scale in preliminary models (Table S2). Averaged across ages 9–17, preterm birth was associated with a 1.1% increase in total problems and a 1.8% increase in emotional problems (compared to term birth). Post-term birth was associated with a 1.2% increase in total problems and a 1.5%, 1.2%, 0.8% and 0.8% increases in emotional, attention/hyperactivity, peer and conduct problems respectively (compared to term birth; Tables S3-7; Fig. S4).

A sensitivity analysis showed that the associations between BW and SDQ scores were slightly attenuated after adjustment for gestational age, however all main effects of BW remained significant (*p* < 0.001). The interaction of BW and time on emotional problems remained a non-significant trend at the corrected p-threshold (*p* = 0.02; Table S10). Similarly, after adjustment for the interaction between gestational age and BW, another small effect attenuation was observed, though with no change in interpretation of main and interactive BW effects (Table S10). The interaction between BW and gestational age was not significant for any SDQ scale at the corrected threshold. A trend interaction was observed for peer problems (*B* = 0.10, *t* = 2.05, *p* = 0.04) such that the association between BW and peer problems was not as strong for preterm births compared to term births (Table S10; Fig. S3).

## Discussion

This is one of the largest longitudinal cohort studies to date on BW, gestational age and childhood mental health. Our findings support the small but significant association between foetal growth and attention and peer problems in childhood. Our findings also support the linear dose–response relationship between foetal growth and multiple aspects of psychopathology across childhood and adolescence. We add several novel observations: (1) the association between restricted foetal growth and attention/hyperactivity problems is relatively stable in magnitude from ages 9 to 17; (2) the association between restricted foetal growth and emotional problems increases throughout adolescence; and (3) the developmental profile of psychopathology linked with foetal growth differs across the sexes.

### Foetal growth and ADHD symptoms

Of all SDQ subscales, the strongest longitudinal effect of foetal growth was on attention/hyperactivity problems. This scale consists of items such as “*[My child is] easily distracted, concentration wanders*” and “*…constantly fidgeting or squirming*”. While this effect size (~ 2% increase in symptoms per kg drop) may seem small, its persistence over time and high replication in other studies [26] supports its robustness.

All foetal growth-linked variation in attention/hyperactivity problems was in the “normal” range, implying even the most restricted foetal growth does not reliably predict clinically-relevant (abnormal/borderline) symptoms (Fig. 1c, Fig. S5). Consistently, Murray et al. [28] found LBW predicted both persistently-mild and partially remitting ADHD symptoms from ages 3 to 14, but not persistently-high symptom levels. Therefore, while foetal growth may show the strongest association with attention/hyperactivity problems, the most clinically-relevant links may be with other aspects of mental health, most notably on late-adolescent peer problems (Fig. 1d).

### Age-dependencies

Several smaller longitudinal studies [17, 19, 38] and a meta-analysis of mostly cross-sectional studies [25] had suggested foetal growth had an increasing association with emotional problems across development. Our study explicitly tested this and showed that (1) reduced foetal growth is linked with increasing emotional problems throughout adolescence, (2) the slope of increase is proportional to the extent of foetal growth restriction, and (3) lower BWs approach the clinically-relevant “borderline” threshold for emotional problems by age 17 (Fig. 1b).

After correction for socioeconomic factors and parental psychiatric history, the interaction between BW and time on emotional problems in this study was not significant at the a-priori p-level. However, the coefficient for the interaction in longitudinal models remained unchanged across all levels of confound adjustment (*B* = − 0.06) therefore attenuation of the effect likely arose from decreasing sample size and increasing variance. Furthermore, cross-sectional models showed that the association between BW and emotional problems was significant at ages 13 and 17, approximately tripling age 9 estimates (Table 2). Finally, the strengthening of the association between BW and emotional problems over time appears to be just beginning in this cohort (Fig. 1b) suggesting this time-dependency may be increasingly evident by the early 20 s.

The psychological correlates of lower BW in childhood may be relatively specific to attention problems, which may in turn increase the risk of internalizing symptoms in adolescence. Another longitudinal study (ages 7–11) suggests that ADHD and internalizing symptoms are reciprocally connected throughout development, with restlessness/hyperactivity a key bridge symptom and excessive worry a strong antecedent for ADHD symptoms [35]. Mediators between foetal growth-linked attention deficits in early childhood and subsequent internalizing problems may include poorer self-esteem [38] or peer problems such as bullying [10].

### Sex differences

Previous studies showed that sex moderated the association between BW and attention problems, with a strong inverse association in males only [12, 27]. In this study, males showed a slightly stronger association between BW and attention/hyperactivity compared to females in sex-stratified models (Tables S8-9) but the interaction was not significant in full-sample models (Table S2). Momany et al. [27] and Dooley et al. [12] were based on U.S. samples therefore differences in our findings may be due to contextual factors differing between the U.S. and Ireland. Our lack of significant sex-by-BW interaction effect on attention problems is consistent with findings of a meta-analysis [26] and Swedish population cohort study [31]. Cross-cohort differences in whether sex moderates the association between BW and child psychopathology need to be explored further to delineate universal/biological effects from context-specific ones.

However, other sex differences were observed. The age-dependent association between BW and emotional problems was stronger in females than males (Fig. 2). The largest psychological correlate of lower BW in males was attention/hyperactivity problems consistently from age 9 to 17 (Table S8), however for females the outcome most associated with BW shifted over time. At age 9, it was peer and attention/hyperactivity problems, at age 13, it was emotional problems and at age 17, it remained emotional problems with a widening lead (Table S9). This may be due to the general increase in emotional problems among females between ages 13 and 17 (Fig. S2), which is consistent with the female rise in depression during this age [3, 6]. It’s also possible this sex difference was driven by a growing under-reporting of emotional problems in males as they age [7, 34]. Our findings are consistent with meta-analytic estimates for LBW on adult depression which is stronger in females (OR = 1.30) than males (OR = 1.12; [11]) and with Yoshimasu et al. [39] who found girls with ADHD were more likely to report internalizing comorbidities than boys.

These findings should be replicated as they have important implications for sex-specific models of psychiatric prediction and treatment.

### Strengths and limitations

Strengths of this study include the use of continuous measures of both BW and mental health allowing us to model a dose–response function. The longitudinal design not only informs developmental theories of mental health, but improves the measurement reliability with 3 SDQ observations available (roughly 4 years apart) for most participants. Generalized mixed models with a gamma distribution modelled the observed distribution of SDQ scores rather than dichotomizing scales to simplify analyses or violating assumptions of normality. Limitations of this study include its reliance on parent reports of both BW and child behaviour (the latter of which may be inaccurate for older adolescents) and that the SDQ does not differentiate between inattention and hyperactivity symptoms, which may have different relationships with birth weight [23]. Finally, the flexibility of our models was somewhat limited by only acknowledging *linear* time within interactions (i.e., time × sex, time × BW) despite the inclusion of a time^2^ main effect.

## Conclusion

Lower BW was associated with increased attention/hyperactivity problems persistently from ages 9 to 17. In males, the longitudinal effect of lower BW was relatively limited to attention/hyperactivity and peer problems, while in females, associations with psychopathology were more widespread. The age-dependent associations between BW and emotional problems should be tested in older longitudinal cohorts, to explore whether this trend continues into adulthood.

### Supplementary Information

Below is the link to the electronic supplementary material.

Supplementary file1 (DOCX 2440 KB)

## Data Availability

The Growing Up in Ireland data is available via application to either the Irish Social Sciences Data Archive or the Central Statistics Office of Ireland depending on the level of detail required. Files containing additional detail and sensitive data (used in this study) are only available via the latter and under strict conditions of confidentiality and data protection.

## Abbreviation

BW: Birth weight
