# Supplementary material for: The persistent effects of foetal growth on child and adolescent mental health: longitudinal evidence from a large population-based cohort
Source: Eur Child Adolesc Psychiatry. 2022 Jul 21;32(10):2067–76. doi: 10.1007/s00787-022-02045-z (PMC10533650; doi:10.1007/s00787-022-02045-z)
Supplement: Supplementary file 1 — Supplementary file1 (DOCX 2440 KB) [file 787_2022_2045_MOESM1_ESM.docx]

Supplementary Material

# Methodological Details

## Equivalized Household Income

An equivalence scale was used to assign a “weight” to each household member (weight of 1 to the first adult in the household, 0.66 to each subsequent adult aged 14+ and 0.33 to each child <14 years). The sum of these weights in each household gives the household’s equivalised size – the size of the household in adult equivalents. Disposable household income was recorded as total gross household income less statutory deductions of income tax and social insurance contributions. Household equivalised income was the disposable household income divided by equivalised household size.

## Preliminary Modelling (M1+)

M1+ included several variations (interactions and polynomials) of the variables of interest (birth weight, gestational age, time, sex) to help establish which fixed effects should be included in subsequent adjusted models. In addition to main effects of sex, time, birth weight and gestational age, the polynomials time^2^ and birth weight^2^ were included as were interactions between time and sex, time and birth weight, birth weight and sex, and gestational age and time.

Results of M1+ (TableS2) showed that the relationship between birth weight and total problems was best captured by a monotonically decreasing line rather than a quadratic form (linear birth weight B= -0.62, SE=0.08, t = -8.20, p< .001; birth weight^2^ B=0.13, SE = 0.07, t=2.02, p=.04). This was also the case most SDQ subscales therefore a birth weight^2^ term was not included in further tests. Similarly, the interaction between sex and birth weight did not have any significant effects on total problems or any subscale so was excluded. The main quadratic effect of time^2^ and the interaction of time *X* sex were retained as they were significant predictors of multiple SDQ scales. Finally, the interaction between time and birth weight, being our hypothesized effect, was retained across all models for theoretical reasons despite showing only a significant effect on emotional problems (TableS2).

# Supplementary Figures

**FigS1:** Histograms of outcome variables: SDQ total problems (top panel) and its 4 subscales.


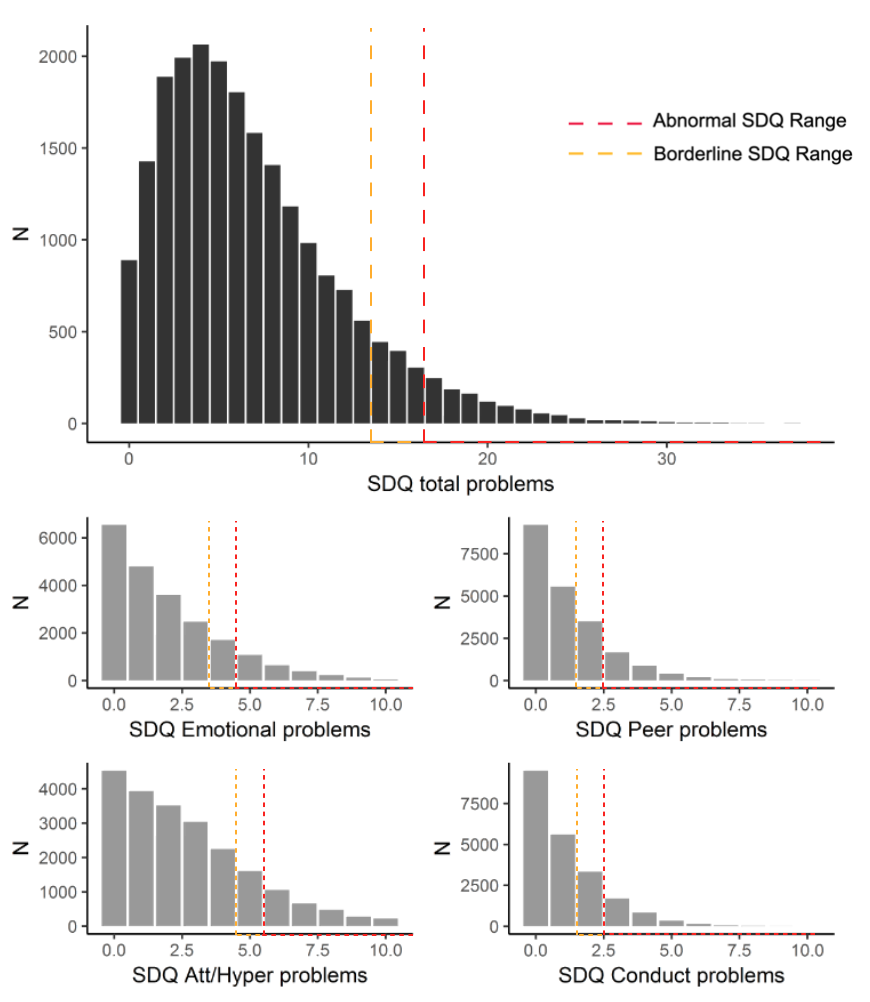


**Note:** Vertical reference lines refer to cut-offs for borderline (yellow) and abnormal (red) scores. All values below “borderline” cut-offs are considered within the “normal” range.

**FigS2.** Descriptive plot of best-fit quadratic association between SDQ scores (y-axis) and time (x-axis), split by sex. Error bands show standard error.


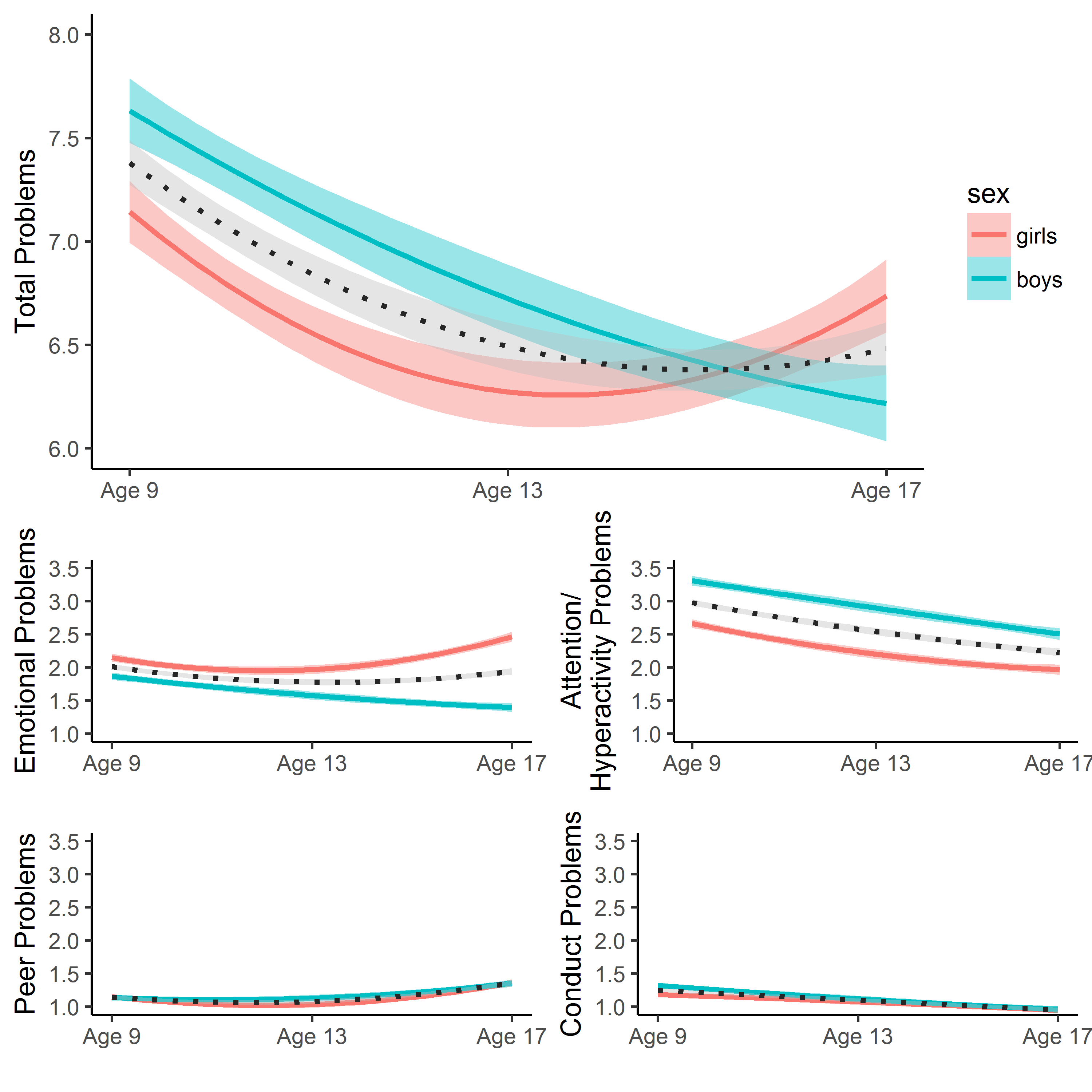

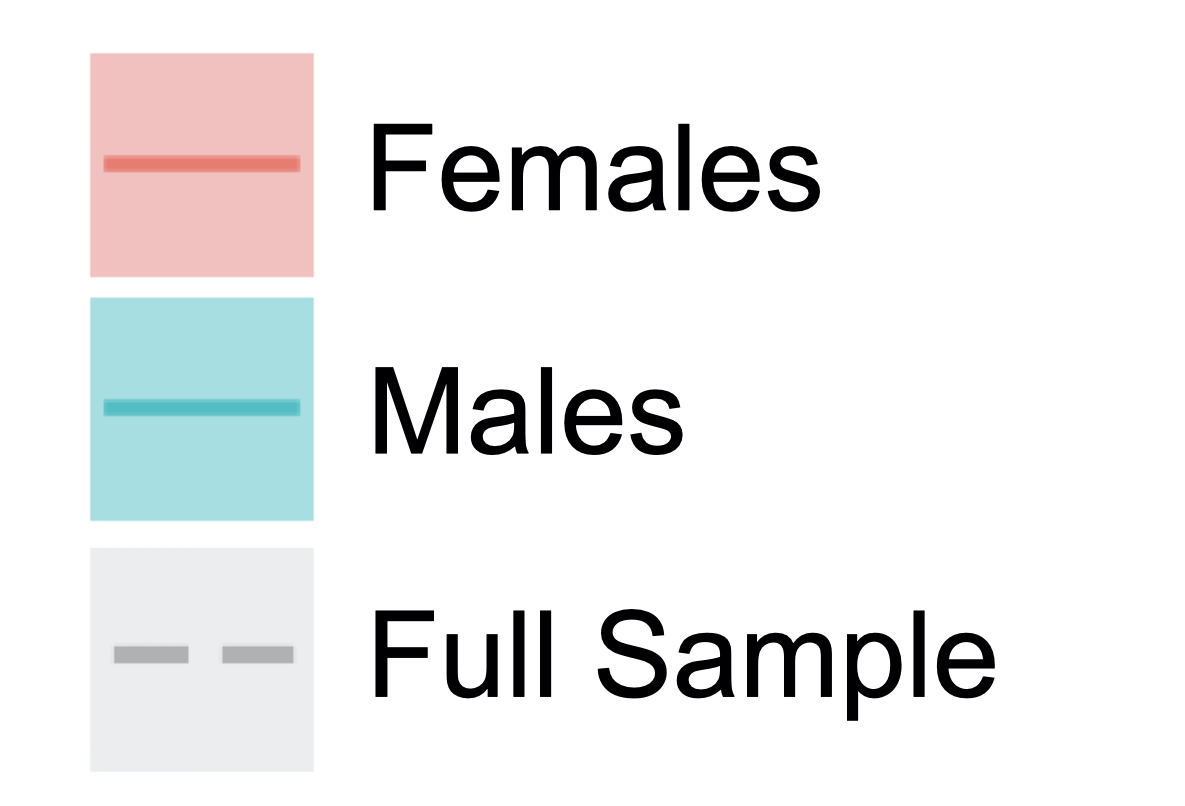


**FigS3.** Descriptive plot showing unadjusted linear association between birth weight (x-axis) and SDQ scales (y-axis), for each gestational age group.


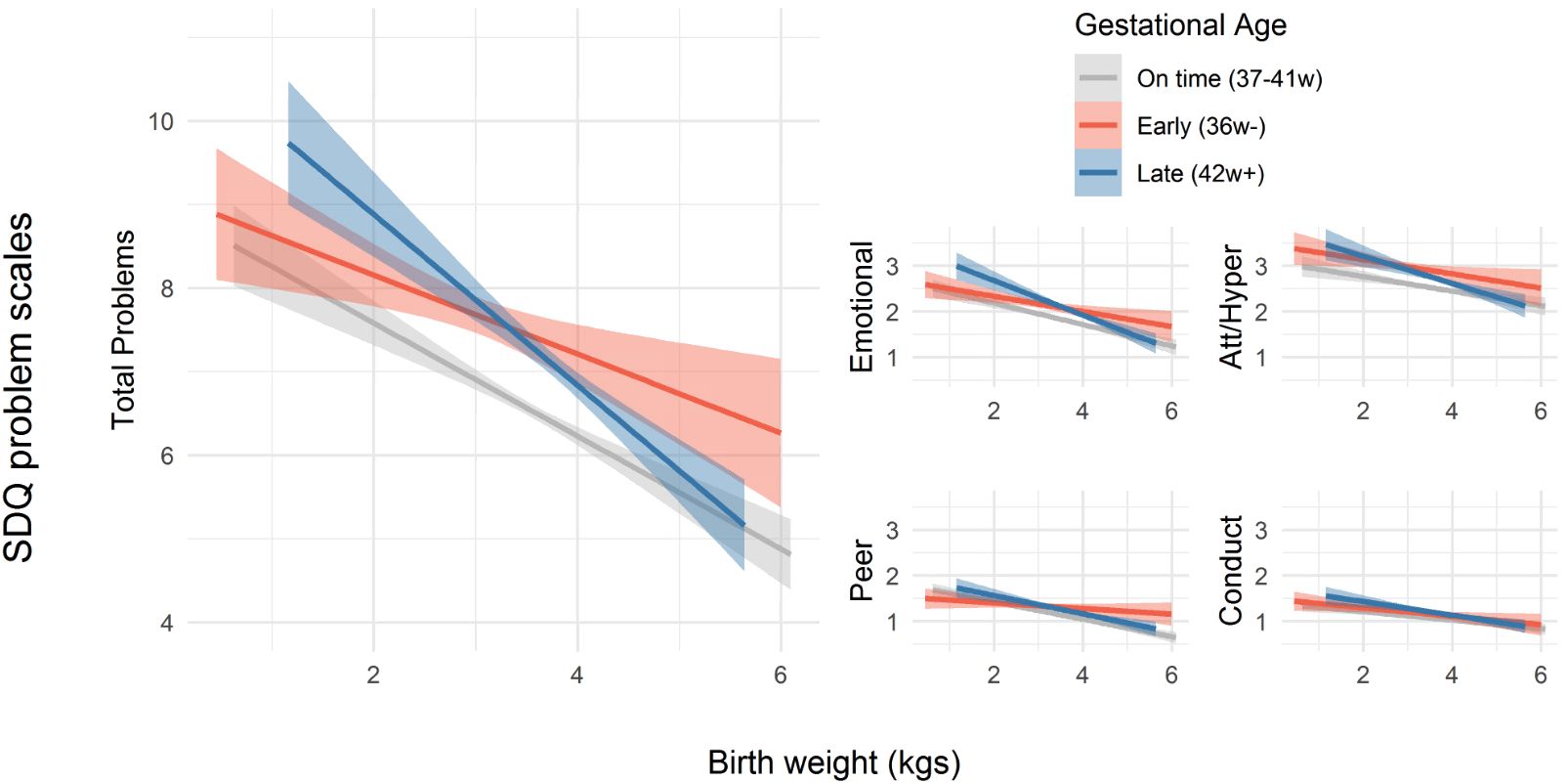


**FigS4.** Fully adjusted association between time (x-axis) and SDQ scales (y-axis) for each gestational age group.


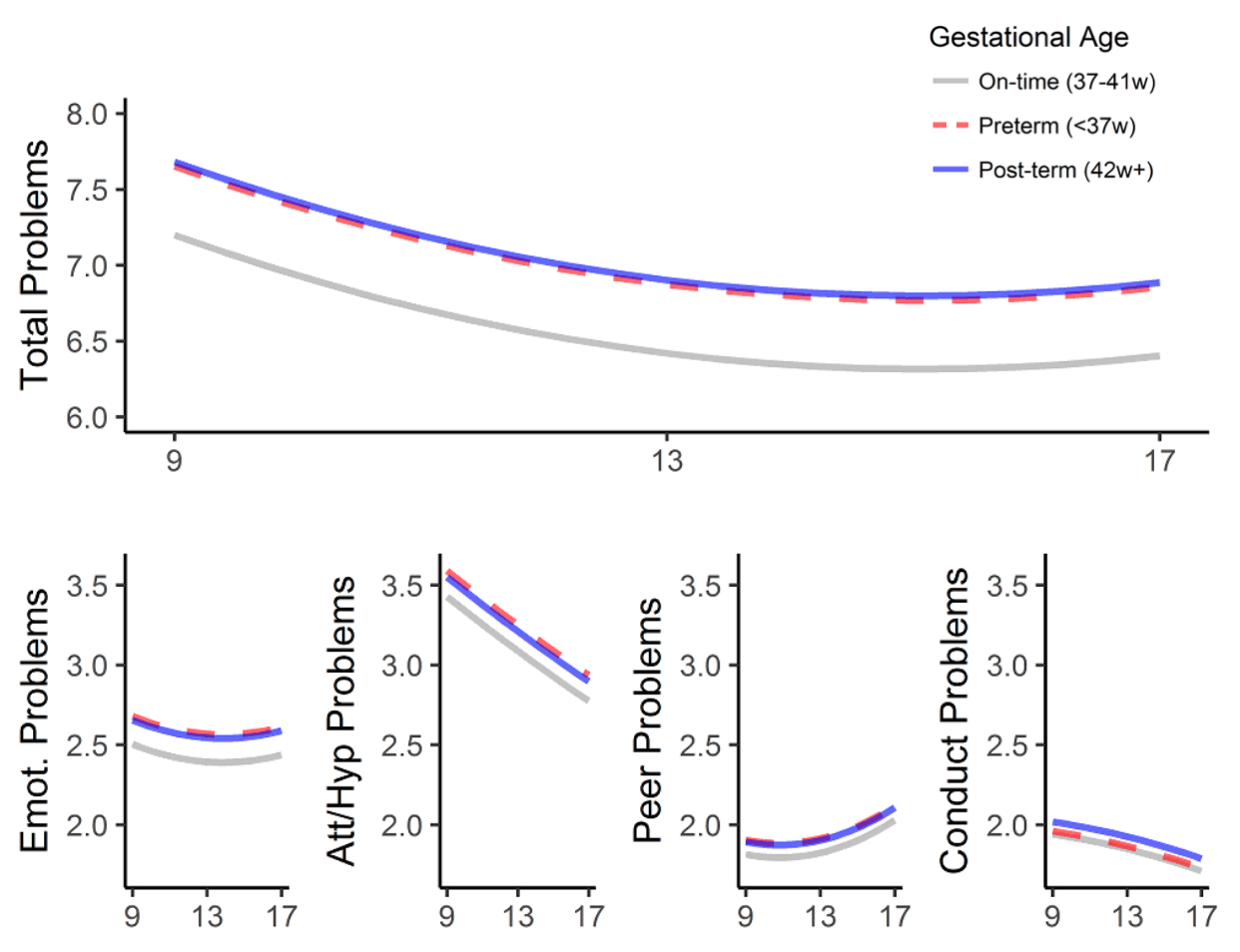


**Note:** Estimates adjusted for sex, birth weight, socioeconomic factors and parental psychiatric history (M3).

**FigS5.** Fully-adjusted association between birth weight groups and SDQ total problems (top panel) and sub-scales (bottom panel) at ages 9, 13 & 17.


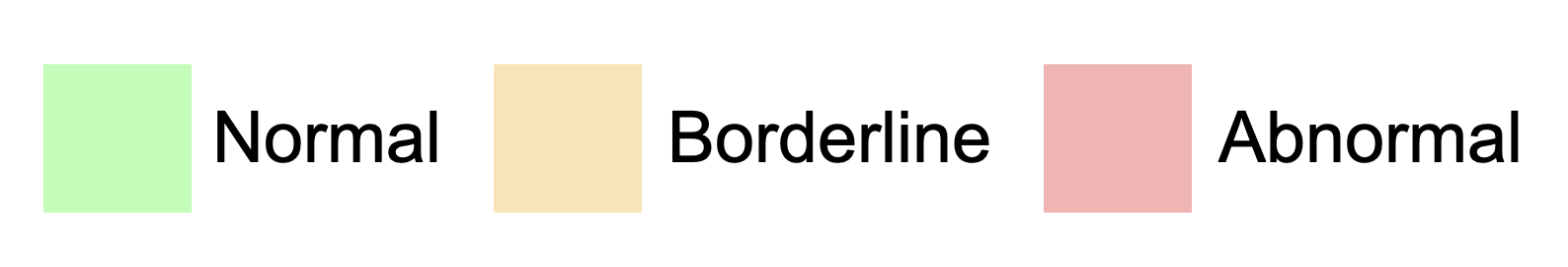

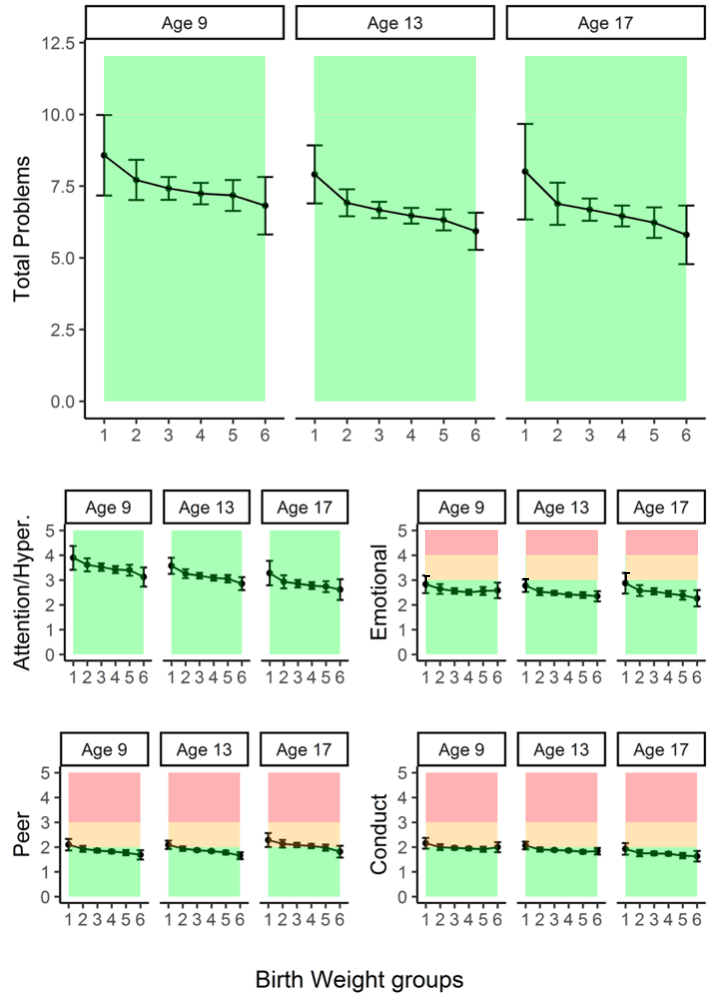


**Note:** Birth weight groups 1-6 refer to the following ranges: (1) <2.50kg, (2) 2.50-2.99kg, (3) 3.0-3.49kg, (4) 3.5-3.99kg, (5) 4.0-4.49kg, (6) 4.5kg or greater. Estimates generated from fully-adjusted longitudinal models (M3). Borderline cut-offs out of range for Total problems (13) & Attention/hyperactivity problems (5).

# Supplementary Tables

**TableS1.** Weighted descriptive statistics. Weighted to compensate for participation and attrition biases based on population-representative distributions of parental education, family structure (e.g., one-parent families), income, social class, child sex and cognitive ability.

|  | Age 9 | | Age 13 | | Age 17 | |
| --- | --- | --- | --- | --- | --- | --- |
| *Categorical Variables* | N | % | N | % | N | % |
| Males | 4220 | 51.0% | 3708 | 50.8% | 3074 | 51.1% |
| Females | 4053 | 49.0% | 3584 | 49.1% | 2946 | 48.9%% |
| Premature born (<37w) | 1013 | 12.4% | 890 | 12.4% | 730 | 12.3% |
| Born late (>41w) | 2112 | 25.9% | 1851 | 25.8% | 1512 | 25.41% |
| Single Parenthood | 1499 | 18% | 1418 | 19% | 1225 | 21% |
| 1 Parent with Mental Illness ^c^ | 1223 | 15% | 918 | 13% | 827 | 14% |
| 2 Parents with Mental Illness ^c^ | 209 | 3% | 237 | 3% | 161 | 3% |
| *Continuous Variables* | Range | M (SD) | Range | M (SD) | Range | M (SD) |
| Birth Weight (kg) | 0.45-6.10 | 3.51 (0.58) | 0.62-6.10 | 3.51 (0.58) | 0.62-6.10 | 3.51 (0.58) |
| Household Income (€)^a^ | 504 - 223K | 19K (13K) | 549 - 134K | 16K (10K) | 504 - 1.2M | 15K (23K) |
| Parental Education level (1-6)^b^ | 1-6 | 3.5 (1.3) | 1-6 | 3.8 (1.3) | 1-6 | 3.7 (1.3) |
| SDQ Total Problems | 0-37 | 7.97 (5.29) | 0-35 | 7.06 (5.41) | 0-33 | 6.92 (5.10) |
| SDQ Attention/Hyperactivity | 0-10 | 3.20 (2.49) | 0-10 | 2.79 (2.46) | 0-10 | 2.42 (2.26) |
| SDQ Emotional | 0-10 | 2.14 (2.05) | 0-10 | 1.90 (2.02) | 0-10 | 2.02 (2.12) |
| SDQ Peer | 0-10 | 1.26 (1.49) | 0-10 | 1.14 (1.49) | 0-10 | 1.43 (1.47) |
| SDQ Conduct | 0-10 | 1.37 (1.50) | 0-10 | 1.23 (1.48) | 0-10 | 1.04 (1.33) |
| 1. Equivalised household income to the nearest thousand (K). Income deciles were used in analysis to limit extreme values. 2. Highest education level attained (highest of both parents taken). Levels 1-6 refer to specific stages of education (see Method). 3. Parent(s) reported a history of depression, anxiety, nerves or other chronic mental health condition (lifetime). | | | | | | |

**TableS2.** M1+ results where additional interactions and polynomials for key variables of interest (birth weight, time, sex) were tested.

|  | Total Problems | | Emotional | | Hyperactivity/Attention | | Peer | | Conduct | |
| --- | --- | --- | --- | --- | --- | --- | --- | --- | --- | --- |
|  | *B*  *(SE)* | *t* | *B (SE)* | *t* | *B*  *(SE)* | *t* | *B*  *(SE)* | *t* | *B*  *(SE)* | *t* |
| Time | -0.36 (0.04) | -9.00** | -0.02 (0.02) | -1.32 | -0.33 (0.02) | -17.49** | -0.12 (0.01) | 9.67** | -0.10 (0.01) | -9.60** |
| Time^2^ | 0.43 (0.03) | 15.16** | 0.08 (0.01) | 6.05** | 0.03 (0.01) | 1.96 | 0.10 (0.01) | 9.62** | -0.02 (0.01) | -1.83 |
| Sex (male) | 0.20 (0.08) | 2.50 ^§^ | -0.45 (0.03) | -14.97** | 0.55 (0.04) | 14.90** | 0.03 (0.02) | 1.18 | 0.05 (0.02) | 2.45 ^§^ |
| Time * Sex | -0.41 (0.07) | -6.29** | -0.28 (0.03) | -10.39** | -0.07 (0.03) | -2.15 ^§^ | 0.01 (0.02) | 0.53 | -0.07 (0.02) | -3.79** |
| Birth Weight (BW) | -0.62 (0.08) | -8.20** | -0.15 (0.03) | -5.24** | -0.22 (0.03) | -6.42** | -0.14 (0.02) | -6.80** | -0.09 (0.02) | -4.61** |
| BW^2^ | 0.13 (0.07) | 2.02^§^ | 0.05 (0.02) | 2.15^§^ | 0.03 (0.03) | 1.06 | 0.01 (0.02) | 0.37 | 0.02 (0.02) | 1.34 |
| Sex * BW | 0.06 (0.14) | 0.41 | 0.07 (0.05) | 1.38 | -0.11 (0.07) | -1.64 | 0.05 (0.04) | 1.39 | 0.03 (0.04) | 0.84 |
| Time * BW | -0.08 (0.06) | -1.26 | -0.07 (0.03) | -2.90* | 0.03 (0.03) | 1.01 | -0.01 (0.02) | -0.59 | -0.01 (0.02) | -0.64 |
| Sex * Time * BW | -0.01 (0.12) | -0.07 | 0.02 (0.05) | 0.35 | 0.04 (0.05) | 0.82 | 0.01 (0.04) | 0.28 | 0.01 (0.03) | 0.22 |
| Late GA | 0.57 (0.09) | 6.11** | 0.17 (0.04) | 4.90** | 0.17 (0.04) | 3.93** | 0.09 (0.03) | 3.50** | 0.10 (0.02) | 4.23** |
| Premature GA | 0.48 (0.14) | 3.51** | 0.17 (0.05) | 3.26* | 0.19 (0.06) | 3.06* | 0.08 (0.04) | 2.30 ^§^ | 0.04 (0.03) | 1.02 |
| Time * Late GA | -0.15 (0.08) | -1.92 | -0.03 (0.03) | -0.88 | -0.03 (0.04) | -0.71 | -0.02 (0.02) | -0.69 | -0.04 (0.02) | -1.95 |
| Time * Premature | -0.21 (0.11) | -1.84 | -0.07 (0.05) | -1.58 | -0.01 (0.05) | -0.24 | -0.05 (0.03) | -1.52 | -0.04 (0.03) | -1.30 |
| Intercept | 6.31 (0.05) | 116.49** | 2.36 (0.02) | 111.66** | 3.06 (0.03) | 119.82** | 1.82 (0.02) | 118.32** | 1.83 (0.01) | 125.07** |
| No. Subjects | 8,180 | | 8,181 | | 8,182 | | 8,183 | | 8,184 | |
| Observations | 21,254 | | 21,281 | | 21,272 | | 21,268 | | 21,278 | |
| AIC | 108,114 | | 69,912 | | 75,576 | | 55,895 | | 52,379 | |
| BIC | 108,257 | | 70,056 | | 75,719 | | 56,038 | | 52,522 | |
| ** *p* < 0.001 **p* < 0.01 ^§^*p* < 0.05  GA: Gestational Age; BW: Birth Weight | | | | | | | |  |  |  |

**TableS3:** Longitudinal prediction of ***Total*** Problems (generalized linear mixed model)

|  | M1 | | M2 | | M3 | |
| --- | --- | --- | --- | --- | --- | --- |
|  | Birth weight, gestational age, sex & time | | M1 + socioeconomic factors | | M2 + parental mental disorder | |
|  | *B (SE)* | *t* | *B (SE)* | *t* | *B (SE)* | *t* |
| Birth Weight (kgs) | -0.63 (0.07) | -8.38** | -0.57 (0.08) | -7.43** | -0.55 (0.08) | -7.14** |
| Time * Birth Weight | -0.07 (0.06) | -1.16 | -0.09 (0.06) | -1.46 | -0.07 (0.06) | -1.11 |
| Time | -0.42 (0.03) | -12.97** | -0.41 (0.03) | -11.87** | -0.40 (0.03) | -11.60** |
| Sex (male) | 0.21 (0.08) | 2.64* | 0.25 (0.08) | 3.15* | 0.25 (0.08) | 3.10* |
| Time * Sex | -0.41 (0.06) | -6.32** | -0.40 (0.07) | -5.77** | -0.40 (0.07) | -5.75** |
| Time^2^ | 0.42 (0.03) | 15.05** | 0.39 (0.03) | 13.08** | 0.38 (0.03) | 12.64** |
| Premature birth (< 37 weeks) | 0.56 (0.13) | 4.24** | 0.45 (0.13) | 3.34** | 0.45 (0.13) | 3.38** |
| Late birth (42 weeks +) | 0.59 (0.09) | 6.34** | 0.50 (0.10) | 5.24** | 0.48 (0.10) | 5.08** |
| Household Income (1-10) |  |  | -0.08 (0.01) | -7.40** | -0.08 (0.01) | -6.81** |
| Parental Education Level (1-6) |  |  | -0.30 (0.03) | -10.19** | -0.30 (0.03) | -10.00** |
| Single-Parenthood |  |  | 0.96 (0.11) | 9.16** | 0.97 (0.11) | 9.14** |
| Parental Psychiatric History |  |  |  |  | 0.76 (0.06) | 11.94** |
| Intercept | 6.34 (0.05) | 123.19** | 6.28 (0.05) | 115.70** | 6.16 (0.05) | 112.12** |
| No. Subjects | 8,180 | | 8,033 | | 8,007 | |
| No. Observations | 21,254 | | 19,559 | | 19,196 | |
| AIC | 108,115 | | 99,224 | | 98,294 | |
| BIC | 108,218 | | 99,350 | | 98,428 | |
| ** *p* < 0.001 **p* < 0.01 ^†^*p* < 0.05.  Gestational age reference group = on-time births (37-41 weeks inclusive). | | | | | |  |

**TableS4:** Longitudinal prediction of ***Attention/Hyperactivity*** Problems (generalized linear mixed model)

|  | M1 | | M2 | | M3 | |
| --- | --- | --- | --- | --- | --- | --- |
|  | Birth weight, gestational age, sex & time | | M1 + socioeconomic factors | | M2 + parental mental disorder | |
|  | *B (SE)* | *t* | *B (SE)* | *t* | *B (SE)* | *t* |
| Birth Weight (kgs) | -0.23 (0.03) | -6.61** | -0. 21 (0.04) | -5.98** | -0.21 (0.04) | -5.80** |
| Time * Birth Weight | 0.03 (0.03) | 0.99 | 0.01 (0.03) | 0.36 | 0.02 (0.03) | 0.58 |
| Time) | -0.33 (0.02) | -22.18** | -0.33 (0.02) | -20.32** | -0.33 (0.02) | -20.08** |
| Sex (male) | 0.55 (0.04) | 14.96** | 0.57 (0.04) | 15.07** | 0.57 (0.04) | 14.98** |
| Time * Sex | -0.06 (0.03) | -2.10 ^§^ | -0.05 (0.03) | -1.43 | -0.05 (0.03) | -1.48 |
| Time^2^ | 0.03 (0.01) | 1.96 | 0.02 (0.01) | 1.05 | 0.01 (0.01) | 0.94 |
| Premature birth (< 37 weeks) | 0.21 (0.06) | 3.39** | 0.16 (0.06) | 2.55^§^ | 0.16 (0.06) | 2.57^§^ |
| Late birth (42 weeks +) | 0.17 (0.04) | 4.00** | 0.13 (0.04) | 2.95* | 0.12 (0.04) | 2.74* |
| Household Income (1-10) |  |  | -0.02 (0.01) | -3.96** | -0.02 (0.01) | -3.40** |
| Parental Education Level (1-6) |  |  | -0.13 (0.01) | -9.46** | -0.13 (0.01) | -9.43** |
| Single-Parenthood |  |  | 0.29 (0.05) | 5.98** | 0.30 (0.05) | 6.14** |
| Parental Psychiatric History |  |  |  |  | 0.18 (0.03) | 6.25** |
| Intercept | 3.06 (0.02) | 126.22** | 3.06 (0.03) | 118.51** | 3.02 (0.03) | 115.55** |
| No. Subjects | 8,180 | | 8,033 | | 8,007 | |
| No. Observations | 21,272 | | 19,574 | | 19,408 | |
| AIC | 75,568 | | 69,529 | | 68,876 | |
| BIC | 75,672 | | 69,655 | | 69,010 | |
| ** *p* < 0.001 **p* < 0.01 ^†^*p* < 0.05.  Gestational age reference group = on-time births (37-41 weeks inclusive). | | | | | |  |

**TableS5:** Longitudinal prediction of ***Peer*** Problems (generalized linear mixed model)

|  | M1 | | M2 | | M3 | |
| --- | --- | --- | --- | --- | --- | --- |
|  | Birth weight, gestational age, sex & time | | M1 + socioeconomic factors | | M2 + parental mental disorder | |
|  | *B (SE)* | *t* | *B (SE)* | *t* | *B (SE)* | *t* |
| Birth Weight (kgs) | -0.13 (0.02) | -6.72** | -0.13 (0.02) | -6.09** | -0.12 (0.02) | -5.79** |
| Time * Birth Weight | -0.004 (0.02) | -0.25 | -0.003 (0.02) | -0.17 | <0.001 (0.02) | 0.001 |
| Time | 0.11 (0.01) | 11.09** | 0.11 (0.01) | 10.09** | 0.11 (0.01) | 10.22** |
| Sex (male) | 0.03 (0.02) | 1.23 | 0.04 (0.02) | 1.92 | 0.04 (0.02) | 1.95 |
| Time * Sex | 0.01 (0.02) | 0.50 | 0.02 (0.02) | 0.85 | 0.02 (0.02) | 0.94 |
| Time^2^ | 0.10 (0.01) | 9.62** | 0.10 (0.01) | 8.82** | 0.10 (0.01) | 8.52** |
| Premature birth (< 37 weeks) | 0.10 (0.03) | 2.83* | 0.08 (0.04) | 2.34 ^§^ | 0.09 (0.04) | 2.47 ^§^ |
| Late birth (42 weeks +) | 0.09 (0.02) | 3.73** | 0.08 (0.03) | 3.12* | 0.08 (0.03) | 3.08* |
| Household Income (1-10) |  |  | -0.02 (0.003) | -5.87** | -0.02 (0.003) | -5.55** |
| Parental Education Level (1-6) |  |  | -0.03 (0.01) | -3.11* | -0.02 (0.01) | -2.83* |
| Single-Parenthood |  |  | 0.20 (0.03) | 6.85** | 0.21 (0.03) | 6.92** |
| Parental Psychiatric History |  |  |  |  | 0.14 (0.02) | 6.91** |
| Intercept | 1.82 (0.01) | 124.53** | 1.80 (0.02) | 115.39** | 1.77 (0.02) | 111.93** |
| No. Subjects | 8,183 | | 8,037 | | 8,007 | |
| No. Observations | 21,268 | | 19,572 | | 19,405 | |
| AIC | 55,888 | | 51,418 | | 50,886 | |
| BIC | 55,992 | | 51,544 | | 51,020 | |
| ** *p* < 0.001 **p* < 0.01 ^†^*p* < 0.05.  Gestational age reference group = on-time births (37-41 weeks inclusive). | | | | | |  |

**TableS6:** Longitudinal prediction of ***Emotional*** Problems (generalized linear mixed model)

|  | M1 | | M2 | | M3 | |
| --- | --- | --- | --- | --- | --- | --- |
|  | Birth weight, gestational age, sex & time | | M1 + socioeconomic factors | | M2 + parental mental disorder | |
|  | *B (SE)* | *t* | *B (SE)* | *t* | *B (SE)* | *t* |
| Birth Weight (kgs) | -0.15 (0.03) | -5.29** | -0.13 (0.03) | -4.50** | -0.12 (0.03) | -4.26** |
| Time * Birth Weight | -0.06 (0.02) | -2.67 * | -0.06 (0.03) | -2.51 ^§^ | -0.06 (0.03) | -2.32 ^§^ |
| Time | -0.04 (0.01) | -2.71* | -0.04 (0.01) | -2.49 ^§^ | -0.04 (0.01) | -2.56 ^§^ |
| Sex (male) | -0.44 (0.03) | -14.85** | -0.43 (0.03) | -14.03** | -0.43 (0.03) | -14.17** |
| Time * Sex | -0.28 (0.03) | -10.42** | -0.28 (0.03) | -9.88** | -0.28 (0.03) | -9.97** |
| Time^2^ | 0.08 (0.01) | 6.04** | 0.08 (0.01) | 6.03** | 0.08 (0.01) | 5.54** |
| Premature birth (< 37 weeks) | 0.20 (0.05) | 4.10** | 0.18 (0.05) | 3.45** | 0.18 (0.05) | 3.45** |
| Late birth (42 weeks +) | 0.18 (0.04) | 5.13** | 0.16 (0.04) | 4.41** | 0.15 (0.04) | 4.18** |
| Household Income (1-10) |  |  | -0.03 (0.005) | -6.22** | -0.03 (0.005) | -5.68** |
| Parental Education Level (1-6) |  |  | -0.09 (0.01) | -7.32** | -0.08 (0.01) | -7.14** |
| Single-Parenthood |  |  | 0.31 (0.04) | 7.51** | 0.29 (0.04) | 7.13** |
| Parental Psychiatric History |  |  |  |  | 0.33 (0.03) | 12.21** |
| Intercept | 2.37 (0.02) | 117.83** | 2.34 (0.02) | 109.68** | 2.29 (0.02) | 106.31** |
| No. Subjects | 8,183 | | 8,037 | | 8,007 | |
| No. Observations | 21,281 | | 19,583 | | 19,416 | |
| AIC | 69,909 | | 64,087 | | 63,446 | |
| BIC | 70,013 | | 64,213 | | 63,580 | |
| ** *p* < 0.001 **p* < 0.01 ^†^*p* < 0.05.  Gestational age reference group = on-time births (37-41 weeks inclusive). | | | | | |  |

**TableS7:** Longitudinal prediction of ***Conduct*** Problems (generalized linear mixed model)

|  | M1 | | M2 | | M3 | |
| --- | --- | --- | --- | --- | --- | --- |
|  | Birth weight, gestational age, sex & time | | M1 + socioeconomic factors | | M2 + parental mental disorder | |
|  | *B (SE)* | *t* | *B (SE)* | *t* | *B (SE)* | *t* |
| Birth Weight (kgs) | -0.09 (0.02) | -4.72** | -0.08 (0.02) | -3.84** | -0.07 (0.02) | -3.70** |
| Time * Birth Weight | -0.01 (0.02) | -0.63 | -0.02 (0.02) | -1.17 | -0.01 (0.02) | -0.83 |
| Time (T1-T3) | -0.12 (0.01) | -13.56** | -0.11 (0.01) | -12.51** | -0.12 (0.01) | -12.63** |
| Sex (male) | 0.05 (0.02) | 2.55 ^§^ | 0.06 (0.02) | 2.86* | 0.06 (0.02) | 2.77* |
| Time * Sex | -0.07 (0.02) | -3.79** | -0.06 (0.02) | -3.45** | -0.06 (0.02) | -3.34** |
| Time^2^ | -0.02 (0.01) | -1.83 | -0.02 (0.01) | -2.01 ^§^ | -0.02 (0.01) | -2.20 ^§^ |
| Premature birth (< 37 weeks) | 0.05 (0.03) | 1.39 | 0.02 (0.03) | 0.59 | 0.02 (0.03) | 0.53 |
| Late birth (42 weeks +) | 0.10 (0.02) | 4.30** | 0.08 (0.02) | 3.19* | 0.08 (0.02) | 3.13* |
| Household Income (1-10) |  |  | -0.02 (0.003) | -6.03** | -0.02 (0.003) | -5.76** |
| Parental Education Level (1-6) |  |  | -0.06 (0.01) | -7.00** | -0.05 (0.01) | -6.84** |
| Single-Parenthood |  |  | 0.13 (0.03) | 4.65** | 0.12 (0.03) | 4.48** |
| Parental Psychiatric History |  |  |  |  | 0.12 (0.02) | 6.61** |
| Intercept | 2.00 (0.04) | 52.94** | 1.98 (0.04) | 49.69** | 1.97 (0.04) | 48.99** |
| No. Subjects | 8,184 | | 8,037 | | 8,007 | |
| No. Observations | 21,278 | | 19,580 | | 19,413 | |
| AIC | 52,374 | | 48,099 | | 46,991 | |
| BIC | 52,478 | | 48,226 | | 47,124 | |
| ** *p* < 0.001 **p* < 0.01 ^†^*p* < 0.05.  Gestational age reference group = on-time births (37-41 weeks inclusive). | | | | | |  |

**TableS8.** Male effects of birth weight on SDQ problems from cross-sectional and longitudinal modelling.

|  | *Cross-sectional effects of birth weight in males^a^* | | | | | |
| --- | --- | --- | --- | --- | --- | --- |
|  | Age 9 | | Age 13 | | Age 17 | |
|  | *B (SE)* | *t* | *B (SE)* | *t* | *B (SE)* | *t* |
| Total Problems | -0.39 (0.15) | -2.62* | -0.49 (0.16) | -3.13* | -0.54 (0.17) | -3.14* |
| Attention/Hyper. Problems | -0.25 (0.07) | -3.40** | -0.21 (0.08) | -2.68* | -0.19 (0.08) | -2.28^†^ |
| Peer Problems | -0.11 (0.04) | -2.70* | -0.11 (0.05) | -2.27^†^ | -0.13 (0.05) | -2.39^†^ |
| Emotional Problems | -0.01 (0.06) | -0.11 | -0.10 (0.06) | -1.67 | -0.10 (0.06) | -1.61 |
| Conduct Problems | -0.03 (0.04) | -0.69 | -0.09 (0.04) | -1.95 | -0.10 (0.05) | -2.21^†^ |
| No. Observations ^b^ | 3644 | | 3255 | | 2572 | |
|  | ***Longitudinal effects of birth weight in males^c^*** | | | | | |
|  | *Birth weight* | | | *Birth weight x Time* | | |
| Total Problems | -0.46 (0.11) | | -4.29** | -0.08 (0.09) | | -0.96 |
| Attention/Hyper. Problems | -0.24 (0.05) | | -4.43** | 0.03 (0.04) | | 0.77 |
| Peer Problems | -0.10 (0.03) | | -3.49** | 0.002 (0.03) | | 0.08 |
| Emotional Problems | -0.06 (0.04) | | -1.54 | -0.05 (0.03) | | -1.47 |
| Conduct Problems | -0.05 (0.03) | | -1.90 | -0.02 (0.02) | | 0.70 |
| No. Observations | 9471 | | | | | |
| ** *p* < 0.001 **p* < 0.01 ^†^*p* < 0.05.  ^a^Adjusted for gestational age at birth, household income, parent education, single-parenthood, parental psychiatric history.  ^b^ There were small differences in number of observations across SDQ scores at age 9 (±11)  ^c^ Adjusted for time, time^2^, gestational age at birth, household income, parent education, single-parenthood, parental psychiatric history. | | | | | | |

**TableS9.** Female effects of birth weight on SDQ problems from cross-sectional and longitudinal modelling.

|  | *Cross-sectional effects of birth weight in females^a^* | | | | | |
| --- | --- | --- | --- | --- | --- | --- |
|  | Age 9 | | Age 13 | | Age 17 | |
|  | *B (SE)* | *t* | *B (SE)* | *t* | *B (SE)* | *t* |
| Total Problems | -0.45 (0.15) | -3.04* | -0.72 (0.15) | -4.66** | -0.83 (0.18) | -4.53** |
| Attention/Hyper. Problems | -0.15 (0.07) | -2.07^†^ | -0.20 (0.07) | -2.82* | -0.21 (0.08) | -2.80* |
| Peer Problems | -0.15 (0.04) | -3.49** | -0.18 (0.05) | -3.84** | -0.19 (0.05) | -3.48** |
| Emotional Problems | -0.12 (0.06) | -1.85 | -0.22 (0.07) | -3.38** | -0.32 (0.08) | -3.76** |
| Conduct Problems | -0.08 (0.04) | -1.87 | -0.15 (0.04) | -3.31** | -0.13 (0.05) | -2.85* |
| No. Observations ^b^ | 3856 | | 3376 | | 2713 | |
|  | ***Longitudinal effects of birth weight in females^c^*** | | | | | |
|  | *Birth weight* | | | *Birth weight x Time* | | |
| Total Problems | -0.64 (0.11) | | -5.83** | -0.06 (0.09) | | -0.61 |
| Attention/Hyper. Problems | -0.18 (0.05) | | -3.62** | -0.001 (0.04) | | -0.03 |
| Peer Problems | -0.14 (0.03) | | -4.80** | -0.003 (0.03) | | 0.11 |
| Emotional Problems | -0.21 (0.05) | | -4.47** | -0.08 (0.04) | | -1.77*^d^* |
| Conduct Problems | -0.10 (0.03) | | -3.40** | -0.01 (0.02) | | -0.50 |
| No. Observations | 9945 | | | | | |
| ** *p* < 0.001 **p* < 0.01 ^†^*p* < 0.05.  ^a^ Adjusted for gestational age at birth, household income, parent education, single-parenthood, parental psychiatric history.  ^b^ There were small differences in number of observations available for each SDQ score at age 9 (±13)  ^c^ Adjusted for time, time^2^, gestational age at birth, household income, parent education, single-parenthood, parental psychiatric history.  ^d^ *p* = 0.08 | | | | | | |

**Table S10.** Testing associations between birth weight and SDQ scores without adjustment for gestational age (1), with adjustment for gestational age (2), and with adjustment for the interaction between birth weight and gestational age (3)

| (1) **Not adjusting** for GA | | | | | | |  | |  |  | |  |  | | |  |  | |  |
| --- | --- | --- | --- | --- | --- | --- | --- | --- | --- | --- | --- | --- | --- | --- | --- | --- | --- | --- | --- |
|  | **BW** | | | **BW x Time** | | |  | |  |  | |  |  | | |  |  | |  |
|  | ***B (SE)*** | ***t*** | ***p*** | ***B (SE)*** | ***t*** | ***p*** |  |  |  |  |  |  |  |  |  | |  |  |  |
| Total | -0.55 (0.07) | -7.60 | <.001 | -0.07 (0.06) | -1.08 | 0.28 |  |  |  |  |  |  |  |  |  | |  |  |  |
| Attention/Hyp. | -0.22 (0.03) | -6.42 | <.001 | 0.02 (0.03) | 0.60 | 0.55 |  |  |  |  |  |  |  |  |  | |  |  |  |
| Peer | -0.12 (0.02) | -6.28 | <.001 | <0.01 (0.02) | 0.01 | 0.99 |  |  |  |  |  |  |  |  |  | |  |  |  |
| Emotional | -0.13 (0.03) | -4.74 | <.001 | -0.06 (0.03) | -2.29 | 0.02 |  |  |  |  |  |  |  |  |  | |  |  |  |
| Conduct | -0.06 (0.02) | -3.43 | <.001 | -0.01 (0.02) | -0.82 | 0.41 |  |  |  |  |  |  |  |  |  | |  |  |  |
| (2) Adjusting for **main effect** of GA | | | | | | | | | | | | |  |  |  | |  |  |  |
|  | **BW** | | | **BW x Time** | | | **Early GA** | | | **Late GA** | | |  |  |  | |  |  |  |
|  | ***B (SE)*** | ***t*** | ***p*** | ***B (SE)*** | ***t*** | ***p*** | ***B (SE)*** | ***t*** | ***p*** | ***B (SE)*** | ***t*** | ***p*** |  |  |  | |  |  |  |
| Total | -0.55 (0.08) | -7.14 | <.001 | -0.07 (0.06) | -1.11 | 0.27 | 0.45 (0.13) | 3.38 | <.001 | 0.48 (0.10) | 5.08 | <.001 |  |  |  | |  |  |  |
| Attention/Hyp. | -0.21 (0.04) | -5.80 | <.001 | 0.02 (0.03) | 0.58 | 0.56 | 0.16 (0.06) | 2.57 | 0.01 | 0.12 (0.04) | 2.74 | 0.006 |  |  |  | |  |  |  |
| Peer | -0.12 (0.02) | -5.80 | <.001 | <0.01 (0.02) | <0.01 | 1.00 | 0.09 (0.04) | 2.47 | 0.01 | 0.08 (0.03) | 3.08 | 0.002 |  |  |  | |  |  |  |
| Emotional | -0.12 (0.03) | -4.26 | <.001 | -0.06 (0.03) | -2.32 | 0.02 | 0.18 (0.05) | 3.45 | <.001 | 0.15 (0.04) | 4.18 | <.001 |  |  |  | |  |  |  |
| Conduct | -0.07 (0.02) | -3.70 | <.001 | -0.01 (0.02) | -0.83 | 0.41 | 0.02 (0.03) | 0.53 | 0.60 | 0.08 (0.02) | 3.13 | 0.002 |  |  |  | |  |  |  |
| (3) Adjusting for **main effect** of GA & **interaction** of GA x BW | | | | | | | | | | | | | | | | | | | |
|  | **BW** | | | **BW x Time** | | | **Early GA** | | | **Late GA** | | | **Early GA * BW** | | | | **Late GA*BW** | | |
|  | ***B (SE)*** | ***t*** | ***p*** | ***B (SE)*** | ***t*** | ***p*** | ***B (SE)*** | ***t*** | ***p*** | ***B (SE)*** | ***t*** | ***p*** | ***B (SE)*** | ***t*** | ***p*** | | ***B (SE)*** | ***t*** | ***p*** |
| Total | -0.57 (0.10) | -5.67 | <.001 | -0.07 (0.06) | -1.10 | 0.27 | 0.56 (0.15) | 3.70 | <.001 | 0.52 (0.10) | 5.14 | <.001 | 0.24 (0.19) | 1.27 | 0.20 | | -0.14 (0.19) | -0.71 | 0.48 |
| Attention/Hyp. | -0.21 (0.05) | -4.39 | <.001 | 0.02 (0.03) | 0.59 | 0.56 | 0.20 (0.07) | 2.81 | 0.005 | 0.14 (0.05) | 2.96 | 0.003 | 0.08 (0.09) | 0.87 | 0.38 | | -0.08 (0.09) | -0.88 | 0.38 |
| Peer | -0.14 (0.03) | -5.27 | <.001 | <0.01 (0.02) | <0.01 | 1.00 | 0.13 (0.04) | 3.15 | 0.002 | 0.08 (0.03) | 3.08 | 0.002 | 0.10 (0.05) | 2.05 | 0.04 | | <0.01 (0.05) | -0.01 | 0.99 |
| Emotional | -0.13 (0.04) | -3.37 | 0.001 | -0.06 (0.03) | -2.31 | 0.02 | 0.19 (0.06) | 3.41 | <.001 | 0.16 (0.04) | 4.16 | <.001 | 0.04 (0.07) | 0.55 | 0.58 | | -0.03 (0.07) | -0.48 | 0.63 |
| Conduct | -0.08 (0.03) | -3.03 | 0.003 | -0.01 (0.02) | -0.82 | 0.41 | 0.04 (0.04) | 0.95 | 0.34 | 0.08 (0.03) | 3.16 | 0.002 | 0.05 (0.05) | 0.93 | 0.35 | | -0.02 (0.05) | -0.37 | 0.71 |

All models are also adjusted for time, time^2^, sex, time *x* sex, household income, single parenthood, parental education and parental psychiatric history.

GA= Gestational age at birth (early [<37 weeks], late [over 41 weeks] or on-time [37-41 weeks])

BW = Birth weight (kilograms)
